# Anti-Arthritic Activity in Rats of Some Phosphinegold(I) Thionucleobases and Related Thiolates

**DOI:** 10.1155/MBD.1998.245

**Published:** 1998

**Authors:** Michael W. Whitehouse, Peter D. Cookson, George Siasios, Edward R. T. Tiekink

**Affiliations:** 1 Department of Medicine The University of Queensland Brisbane Queensland 4072 Australia; 2 Department of Pathology The University of Adelaide 5005 Australia; 3 Department of Chemistry The University of Adelaide 5005 Australia

## Abstract

A number of phosphinegold(I) thiolates where, generally, the thiolate is derived from a thionucleobase, have been screened for anti-arthritic activity in Dark Agouti rats, a gold sensitive model for arthritis. Potency and toxicity data showed that, generally, the Ph_3_P derivatives and species based on thiopurines were the most effective and that with other complexes enhanced activity was accompanied by greater toxicity.


### ANTI-ARTHRITIC ACTIVITY IN RATS OF SOME PHOSPHINEGOLD(I) THIONUCLEOBASES AND RELATED THIOLATES

Michael W. Whitehouse[1,2], Peter D. Cookson[3], George Siasios[3], and Edward R.T. Tiekink[3*]

[1] Department of Medicine, The University of Queensland, Brisbane, Queensland 4072, Australia
[2] Department of Pathology, The University of Adelaide, Australia 5005
[3] Department of Chemistry, The University of Adelaide, Australia 5005

**Abstract**

A number of phosphinegold(I) thiolates where, generally, the thiolate is derived from a thionucleobase, have been screened for anti-arthritic activity in Dark Agouti rats, a gold sensitive model for arthritis. Potency and toxicity data showed that, generally, the $Ph_3P$ derivatives and species based on thiopurines were the most effective and that with other complexes enhanced activity was accompanied by greater toxicity.

**Introduction**

Aurothiolates, such as sodium aurothiomalate (Myocrisin) and aurothioglucose (Solganol) (Figure 1), have been used for over 70 years to treat rheumatoid arthritis (RA) and may have potential as anti-cancer (anti-tumour) agents [1-4]. Quite surprisingly, though potent and effective, little systematic research has been published relating anti-arthritic activity (in experimental animals) to the chemical structure of gold(I) complexes since the development (1971) of Auranofin ([(1-thio-β-D-glucopyranose-2,3,4,6-tetraacetato-S)(triethylphosphine)-gold(I)], Figure 1), an orally active gold(I) complex with both thio and phosphine ligands.

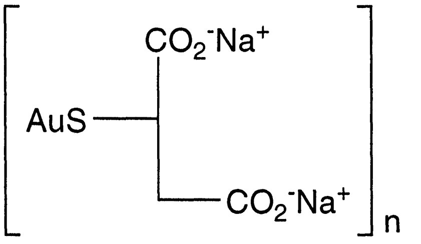

Myocrisin

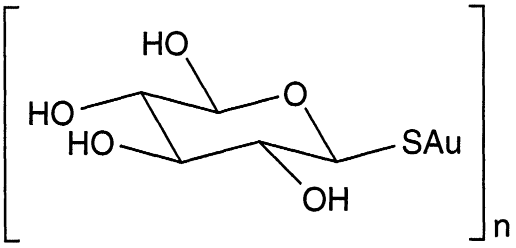

Solganol

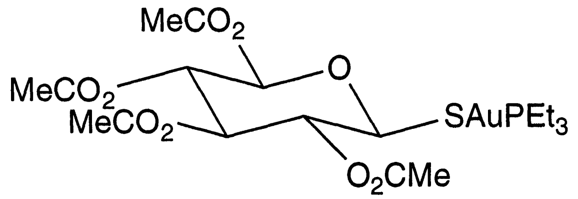

Auranofin

Figure 1. Chemical structures of some gold drugs used in the treatment of RA.

The perceived side-effects (renal, enteric, dermal), delayed action and variable bio-availability of the currently available hydrophilic Myocrisin and lipophilic Auranofin drugs have been viewed as a deterrent, rather than as a stimulus to search for alternative gold(I) drugs. Yet, despite massive investments in research into other classes of immunoregulants

(biological or synthetic) over the past 25 years, there is no anti-arthritic drug available apart from Myocrisin which has been deemed curative (*i.e.* inducing remission) for RA rather than just disease suppressant.

This report summarises some of our findings relating chemical structure, based on the P-Au-S atomic arrangement as found in lipophilic Auranofin, to anti-arthritic activity in an animal model. The choice of thiolate is dictated by the fact that many of the chosen thionucleobases have biological activity themselves [*e.g.* 5]. Further, a number of species reported in this contribution have proved to possess significant *in vitro* cytotoxicity [6,7].

### Materials and methods

### *Synthesis*

The complexes were prepared according to established procedures: 6-mercaptopurine complexes (6-MP) [8], 6-thioguanine (6-TG) [7], 2-thiouracil (2-TU) [9], 5-carboxy-2-thiouracil (5-$CO_2$-TU) [9,10], 6-methyl-2-thiouracil (6-Me-2-TU) [11], 6-nPr-2-thiouracil (6-nPr-2-TU) [12,13], 2-mercaptopyridine (2-pyS) [14], 2-mercaptopyrimidine (2-pymS) [14], *O*-Alkyldithiocarbonate ($S_2COR$) [15-17] and [$Au(SC(NPh)PPh_2)_2$] [18]. Figure 2 shows the chemical formulae of the thionucleobases and analogues used in this study.

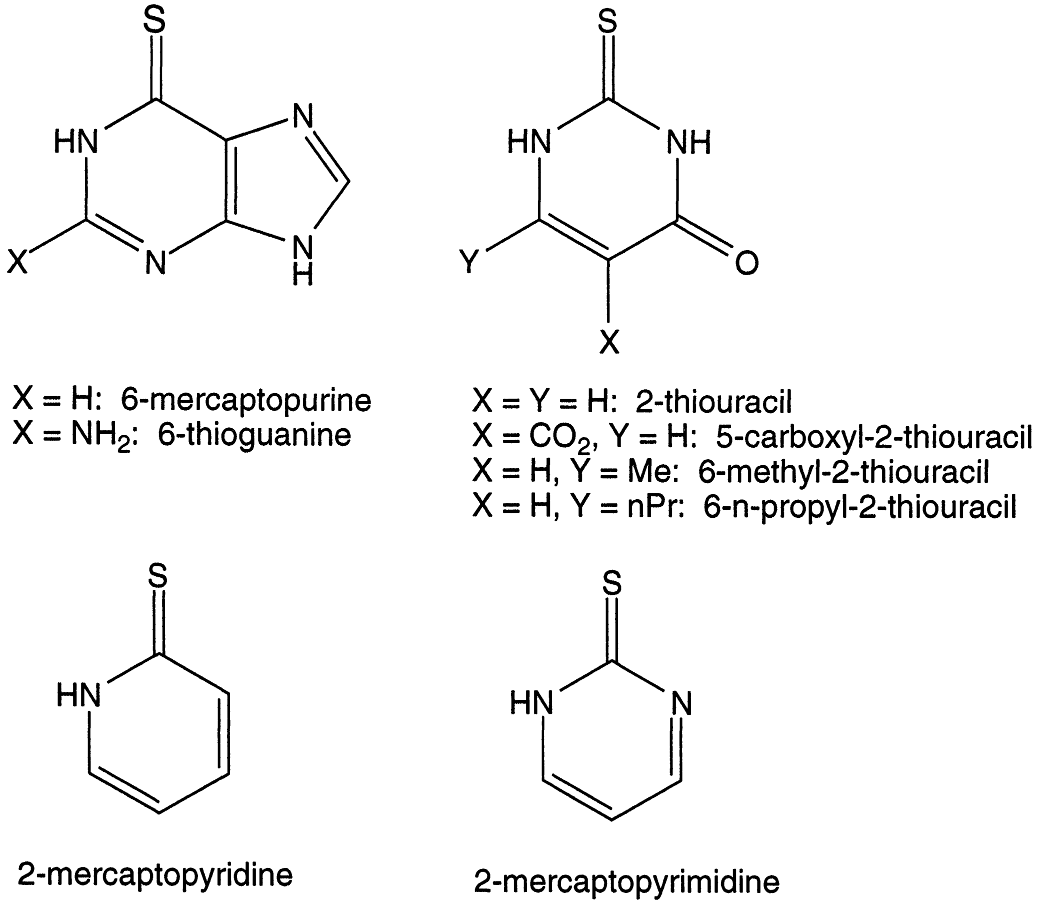

Figure 2. Chemical structure of some of the thiols used in this study.

### *Anti-arthritic screening*

Screening for anti-arthritic activity was performed by examining the response to the gold complexes in female dark agouti (DA) rats, a gold-sensitive rat strain [19]. DA rats were dosed on alternate days (total doses = 7) given i.m., s.c. or p.o. as dispersions in 0.02% Tween-20-saline or applied dermally in DMSO or DMF. Arthritis was engendered with a mycobacterial adjuvant injected into the tail base and expressed in all 4 paws and in tail joints after 14 days. The maximum doses were < 10 mg Au/kg. Sodium aurothiomalate (6 mg Au/kg) given s.c. or Auranofin (6 mg Au/kg) given p.o. were used as reference complexes.

### Results and Discussion

### *Screening protocol*

The screening for anti-arthritic activity involved the inducement of an autoallergic polyarthritis by the injection of an arthritogenic Freund's adjuvant on day 0. Figure 3 shows the lower parts of three animals. The one on the left is an healthy animal and the one on the right has contracted arthritis as seen in the paw and tail swelling. The middle animal has been treated with a gold complex: evidence for reduced inflammation can be noted from the reduced swelling in the paws and tail, indicating the influence of the gold complex on the arthritis. In Table I ratings for both toxicity and anti-arthritic potency are given. Toxicity was assessed by weight loss, diarrhoea and albuminuria in both normal and arthritic rats. A score of 2+ was considered as unacceptable. Treatments completely suppressing polyarthritis on day 18 (*i.e.* four days after last dose) were scored at 3+. A rating of 4+ indicated that no arthritis had developed by day 28.

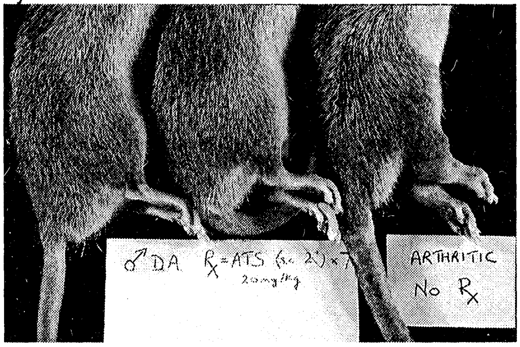

Figure 3. Lower regions of three dark Agouti rats, from left to right: healthy animal; arthritic animal treated with gold drug; arthritic animal with no treatment.

### *Anti-arthritic activity*

The results of the anti-arthritic screening for a number of phosphinegold(I) thiolate complexes are listed in Table 1.

Several conclusions may be drawn from the experimental results present in Table I. Clearly, several of the listed monomeric phosphinegold(I) thiolate complexes, where the thiolate ligand is derived from a thionucleobase analogue, show comparable if not greater potency as well as reduced toxicity when compared to the clinically used drugs, Myocrisin and Auranofin.

Of the complexes listed in Table I, the most active and least toxic species is [$Et_3PAu$(6-MP)]. Of the thiolates, the 6-mercaptopurinates (6-MP) tend to have the greater potency. It should be noted here that 6-mercaptopurine and related thionucleobases possess anti-arthritic activity in their own right [5] but that the addition of $R_3PAu$ leads to increased potency [8]. The addition of an amino substituent at C-2 in 6-MP, leading to 6-TG, generally reduces the potency and increases the toxicity. It is of interest that in the 6-MP series of complexes, it is the $Et_3P$ derivatives that possess the greater activity and lesser toxicity than the other $R_3P$ analogues noting that it is $Et_3P$ that is found in Auranofin. This result contrasts that found in subsequent series where both $Ph_3P$ and $Cy_3P$ appear to have the greater potential (see below).

Considering next the 2-thiouracilate complexes, substitution in the ring tends to increase the potency but toxicity for these derivatives was a problem, with their administration leading to unhealthy animals. Although not thionucleobases, some 2-mercaptopyridine and 2-mercaptopyrimidine (2-pym) derivatives were also tested. Of these, the 2-pym complex was the more active, however, toxicity was again a problem.

Table I: Potential therapeutic activity in rats[a]

| | Complex | Toxicity | Anti-arthritic potency | Ref. |
|---|---|---|---|---|
| **1** | [$Et_3PAu(6\text{-}MP)$] | - | 4+ | [8] |
| **2** | [$Ph_3PAu(6\text{-}MP)$] | + | 3+ | [8] |
| **3** | [$Cy_3PAu(6\text{-}MP)$] | + | 4+ | [8] |
| **4** | [$Et_3PAu(6\text{-}TG)$] | + | 2+ | |
| **5** | [$Ph_3PAu(6\text{-}TG)$] | 2+ | 4+ | |
| **6** | [$Cy_3PAu(6\text{-}TG)$] | + | 3+ | |
| **7** | [$Et_3PAu(2\text{-}TU)$] | not available | 0 | [9] |
| **8** | [$Ph_3PAu(2\text{-}TU)$] | not available | 2+ | [9] |
| **9** | [$Cy_3PAu(2\text{-}TU)$] | 2+ | + | |
| **10** | [$Ph_3PAu(5\text{-}CO_2\text{-}2\text{-}TU)$] | 2+ | 4+ | |
| **11** | [$Cy_3PAu(5\text{-}CO_2\text{-}2\text{-}TU)$] | 0 | 3+ | |
| **12** | [$Ph_3PAu(6\text{-}Me\text{-}2\text{-}TU)$] | + | 3+ | |
| **13** | [$Ph_3PAu(6\text{-}nPr\text{-}2\text{-}TU)$] | 2+ | 4+ | |
| **14** | [$Cy_3PAu(6\text{-}nPr\text{-}2\text{-}TU)$] | + | 2+ | |
| **15** | [$Ph_3PAu(2\text{-}pyS)$] | + | 0 | |
| **16** | [$Ph_3PAu(2\text{-}pymS)$] | 2+ | 4+ | |
| **17** | [$Cy_3PAu(2\text{-}pymS)$] | 2+ | 3+ | |
| | Sodium aurothiomalate | - | 2+ | |
| | Auranofin | 2+ | 2+ | |

a: see text for explanation of ratings

Preliminary screening was also conducted on some related phosphinegold(I) thiolates but these were not as promising as some of the complexes tabulated in Table I and, hence, full toxicity screens were not conducted. Several phosphinegold(I) dithiocarbonates (Figure 4) were tested. For $R_3PAu(S_2COMe)$, the potency was rated as + and 3+ for R = Ph and Cy, respectively; the R = Et complex was lethal, however, for $R_3PAu(S_2COiPr)$ the ratings were 3+ and 0 for R = Ph and Cy, respectively. Finally, for the dinuclear complex, $[Au(SC(=N)PPh_2)]_2$ (incorporating S- and P- donors in the one ligand, Figure 4), the potency and toxicity was scored at + and 2+, respectively.

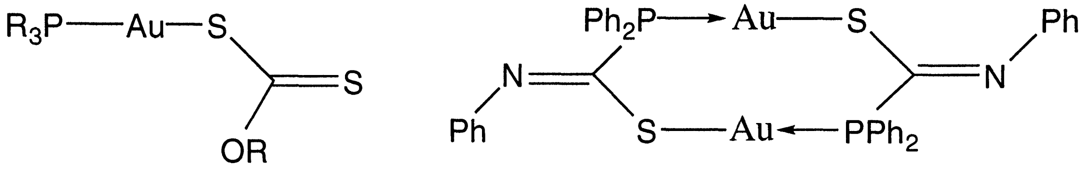

R = Et, Ph, Cy
R' = Me, iPr

Figure 4. Chemical structures for $R_3PAu(S_2COiPr)$ [15-17] and $[Au(SC(=N)PPh_2)]_2$ [18].

***Conclusions***

The biological data indicate that some of phosphinegold(I) thiolates where the thiolate is derived from a thionucleobase, *i.e.* systems analogous to Auranofin, possess significant potency against an induced arthritis in the Dark Agouti rat model. These activities rivalled those found for both Myocrisin and Auranofin. Further, some of these derivatives were less toxic than the commercially available gold complexes. Structure correlations suggested that while the most active complex contained the $Et_3P$ ligand, generally $Ph_3P$ derivatives tended to be more active. That there is not a definitive relationship between potency/toxicity and either the nature of the phosphine and thiolate ligand in the systems studied indicates that the biological activity must arise from a subtle interplay between both the phosphine and thiolate ligands.

**Acknowledgments**
We thank the Australian Research Council and The University of Adelaide for supporting these studies.

**References**

1 A.J. Lewis, D.T. Walz, *Prog. Med. Chem.* 1982, **19**, 1.
2 R.V. Parish, *Interdisciplinary Sci. Rev.*, 1992, **17**, 221.
3 P.J. Sadler, R.E. Sue, *Metal-Based Drugs* 1994, **1**, 107.
4 E.R.T. Tiekink and M.W. Whitehouse, in *Handbook of Metal-Ligand Interactions in Biological Fluids.* G. Berthon (Ed.) Marcel Dekker, Inc., New York, Vol. 2, 1995, 1266.
5 J.J. McCormack, D.G. Johns, in *Cancer Chemotherapy: Principles and Practice*, B.A. Chabner, J.M. Collins (Eds) Lippincott Company, Philadelphia, 1990, Chapter 9.
6 E.R.T. Tiekink, P.D. Cookson, B.M. Linahan, L.K. Webster, *Metal-Based Drugs* 1994, **1**, 299.
7 L.K. Webster, S. Rainone, E. Horn, E.R.T. Tiekink, *Metal-Based Drugs* 1996, **3**, 63.
8 P.D. Cookson, E.R.T. Tiekink, M.W. Whitehouse, *Aust. J. Chem.* 1994, **47**, 577.
9 C.S.W. Harker, E.R.T. Tiekink, M.W. Whitehouse, *Inorg. Chim. Acta* 1991, **181**, 23.
10 P.D. Cookson, B.Sc.(Hons) report, The University of Adelaide, 1991.
11 P.D. Cookson, E.R.T. Tiekink, *J. Chem. Cryst.* 1994, **24**, 805.
12 P.D. Cookson, E.R.T. Tiekink, *Z. Kristallogr.* 1994, **209**, 749.
13 P.D. Cookson, E.R.T. Tiekink, *J. Cryst. Spectros. Res.* 1993, **23**, 231.
14 P.D. Cookson, E.R.T. Tiekink, *J. Chem. Soc., Dalton Trans* 1993, 259.
15 E.R.T. Tiekink, *Z. Kristallogr.* 1985, **173**, 243.
16 G. Siasios, E.R.T. Tiekink, *Z. Kristallogr.* 1992, **198**, 139.
17 G. Siasios, E.R.T. Tiekink, *Z. Kristallogr.* 1993, **205**, 261.
18 D. Clajus, R. Kramolowsky, G. Siasios, E.R.T. Tiekink, *Inorg. Chim. Acta*, in press.
19 I.R. Garrett, M.W. Whitehouse, B. Vernon-Roberts, P.M. Brooks, *J. Rheumatol.*, 1985, **12**, 1079.

**Received: July 8, 1998 - Accepted: July 14, 1998**

